# Cyclists’ exposure to air pollution, noise, and greenery: a population-level spatial analysis approach

**DOI:** 10.1186/s12942-023-00326-7

**Published:** 2023-02-10

**Authors:** Elias Willberg, Age Poom, Joose Helle, Tuuli Toivonen

**Affiliations:** 1 Digital Geography Lab, Faculty of Science, University of Helsinki, Helsinki, Finland; 2 Mobility Lab, Department of Geography, University of Tartu, Tartu, Estonia; 3 Helsinki Institute of Sustainability Science, Institute of Urban and Regional Studies, University of Helsinki, Helsinki, Finland

**Keywords:** Environmental exposure, Active travel, Route choice, Air pollution, Noise, Greenery

## Abstract

Urban travel exposes people to a range of environmental qualities with significant health and wellbeing impacts. Nevertheless, the understanding of travel-related environmental exposure has remained limited. Here, we present a novel approach for population-level assessment of multiple environmental exposure for active travel. It enables analyses of (1) urban scale exposure variation, (2) alternative routes’ potential to improve exposure levels per exposure type, and (3) by combining multiple exposures. We demonstrate the approach’s feasibility by analysing cyclists’ air pollution, noise, and greenery exposure in Helsinki, Finland. We apply an in-house developed route-planning and exposure assessment software and integrate to the analysis 3.1 million cycling trips from the local bike-sharing system. We show that especially noise exposure from cycling exceeds healthy thresholds, but that cyclists can influence their exposure by route choice. The proposed approach enables planners and individual citizens to identify (un)healthy travel environments from the exposure perspective, and to compare areas in respect to how well their environmental quality supports active travel. Transferable open tools and data further support the implementation of the approach in other cities.

## Introduction

Environmental exposures are linked with diverse health and wellbeing impacts that shape liveability in urban areas [1–3]. Air pollution resulting from human activity is the single largest environmental health risk, with around 400 000 premature deaths per year in Europe [4] and 91% of the global population lack access to clean air [5]. Other exposures such as noise [6], heat [7], toxic chemicals [8], or heavy metals [9] also have significant negative health impacts in urban areas. In contrast, some other daily exposures can support human health and well-being. Urban greenery, meaning urban parks, forests, and street trees, has been linked to positive impacts through various pathways including reducing the harm of negative exposures, restoring mental capacities, and encouraging physical activity [10]. One of the main sources of daily exposure is everyday travel, which exposes people to multiple and dynamic environmental variables [11]. On a strategical level, access to safe, healthy, and sustainable urban transport is recognised among the UN's Sustainable Development Goals [12] and is a key goal for urban and transportation planning [13]. However, mounting evidence from real life shows that we are still far from the strategic goals as the burdens of negative exposures and the opportunities for health-supporting positive exposures resulting from travel are unequally distributed between areas, demographic groups, and travel modes [2, 14–16].

Travel-related environmental exposures are especially relevant in the active travel context. Walking and cycling have well-known health benefits resulting from physical activity [e.g. 17], but those who walk or cycle are also more directly in contact with their surrounding environment and might therefore receive a higher exposure dose than those who use motorised modes of travel [18, 19]. Urban and transportation planning now recognises active travel as a key component in urban areas for improving the health of urban residents, arranging human mobility efficiently within limited urban street space, mitigating the environmental burden of transport and responding to climate change [20]. There is also more awareness among planners of the negative health impacts of environmental exposure, and consequently a growing need to identify areas in which exposures exceed safe levels for health [20]. The travel environment quality is particularly important for encouraging *more* active travel, as people are likelier to walk and cycle in environments that are attractive, pleasant, and safe [21–23]. Ensuring access to these environments helps to reduce the determinants of socio-spatial health inequalities [2].

Currently, the understanding of environmental exposures during travel, the linkages between multiple and cumulative exposures, and their health and well-being impacts is still inadequate [11]. A large body of literature has examined environmental exposures from the residential perspective, but less attention has been directed to the exposure during daily travelling, even if it contributes a significant proportion of typical daily exposure [24–26]. It is documented that ignoring exposure from travel may lead to both over- and underestimations in exposure assessments, depending on contextual aspects [24, 26]. The knowledge gap in travel-related exposure assessment is a result of several conceptual and methodological challenges. In this study, we focus on two methodological challenges, which are identified by previous literature. These are the questions how to extrapolate travel-related exposure measures to the population level that is relevant for spatial planning [16, 27] and how to assess the associations between multiple travel-related exposures at this level [28–30].

Extrapolation of travel-related exposure measures to the level of populations requires representative mobility data. They should show realised travel behaviour of people over a sufficiently long period and broadly cover different parts of the urban fabric to represent mobility structures. While many exposure studies have used for example GPS tracks [e.g. 19, 31, 32] or travel surveys [24, e.g. 33] for capturing mobility patterns, these types of datasets are rarely available with such spatio-temporal coverage, which is needed for population-level analyses. In this respect, the rapid emergence and popularity of bike-sharing systems in many cities has provided new opportunities. These systems typically cover large territories of their cities and serve most of the year, producing as a by-product spatially representative mobility data on cycling for population-level assessments [34, 35]. Similarly, advances in the collection of environmental data have provided potential to focus on less studied exposure types, for example on greenery and noise levels [36, 37]. Improved access to environmental data has also provided opportunities to evaluate how different travel-related exposure types might amplify or balance the impacts of each other [38]. In addition, the development of environmentally sensitive route-planning tools has supported population-level and multiple exposure assessments from travel by enabling researchers to assess the individual exposure load from certain routes and compare them with alternatively chosen routes [39–41].

Drawing from these advances, in this study, we present a novel approach for population-level assessment of multiple environmental exposure from active travel. Our approach enables to (1) reveal the spatial distribution of air pollution, noise, and greenery exposure based on realised cycling behaviour data at urban scale; (2) compare the potential to improve the exposure load by using alternative route choice, and (3) compare the spatial similarity of exposure-optimised routes to understand whether alternative route choice can yield multiple exposure benefits. These elements have significant value for spatial planners, individual citizens and researchers as they help them to understand and mitigate negative health impacts from travel with individual and collective action and to prioritise development needs. The approach also helps to assess how well the environmental quality in different neighbourhoods supports active travel and to connect this information with realised travel behaviours of people. Transferable open tools and data employed by the approach further support the implementation in other cities. We demonstrate the feasibility of the approach in Helsinki using an in-house developed Green Paths Software [42]. Finally, we discuss the implications of the findings and the applicability of the approach and outline steps for future research.

## Background

### Air pollution and cycling

A large body of literature has examined the connections between air pollution exposure and cycling. Most studies focus on particulate pollutants such as PM_10_, PM_2.5_, UFP, PNC^1^ and Black Carbon, but exposure to gas pollutants such as Carbon and Nitrogen Oxides and Volatile Organic Compounds has also been studied [30]. In comparison to other travel modes, cyclists have consistently been found to inhale larger doses of pollutants, as summarised by several reviews [30, 43–45]. The larger inhaled dose is largely a result of increased inhalation during cycling and longer trip times, since evidence does not typically suggest higher exposure levels for cyclists. Many studies have found cyclists to be subjected to lower exposure levels, [43, 44] while a few studies have found that cyclists are subjected to similar exposure levels [19] or higher levels [46] of exposure to air pollution compared to motorised modes. However, significant methodological variations in exposure assessment and the heterogeneity of travel settings between the studies in the field still limit generalisable conclusions [43]. Results on short-term health impacts on pulmonary functions, cardiac functions, inflammation, and stress resulting from air pollution exposure remain mixed regardless of the study setting [47–50]. The studies examining long-term impacts often aim to estimate the net impact of air pollution exposure from cycling on mortality or morbidity [51, e.g. 52]. These studies generally agree that despite air pollution exposure posing a significant health risk, the net benefits of cycling to health almost always outweigh the negative impacts of air pollution [3, 17, 51]. In relation to route choice, cyclists are keen on avoiding the most polluted cycling routes by choosing a less polluted route option, but there are differences between cyclist groups [53–55]. Nevertheless, air pollution does not seem to have an impact on cycling frequency [33]. However, the perception of air pollution exposure might differ from the measured dose with cyclists underestimating their realised exposure [56, 57].

### Noise and cycling

Despite the overall health effects of noise, exposure to noise during cycling has remained an understudied topic [30]. Studies from Delhi, Montreal and from eleven Dutch cities found that the cyclists were exposed to excessive noise in these cities [58–60]. In one study, noise exposure of cycling was found to be the highest along major roads and downtown [31]. Compared to other travel modes, cyclists were found to be exposed to higher noise levels in some studies [61, 62]. However, due to the variety of methodologies and ways to measure noise exposure and travel details, as well as the small number of studies on noise exposure and cycling, strong conclusions cannot be drawn [30]. Noise does not seem to be a major obstacle to the overall propensity to cycling [30], but it has been found to have an impact on route choice. Cyclists in Germany and Austria were willing to travel on routes that were 6.4% longer to avoid traffic impacts, including noise pollution [54]. Routes away from the traffic noise was also the most significant motivator for the respondents in Vancouver, increasing the likelihood of cycling [63]. Cyclists may also perceive their noise exposure to be significantly lower than is measured [56, 57], but there are individual differences in the perception of noise [64]. Understanding how noise exposure from cycling influences health and well-being in the short term has remained extremely limited. Buregeya et al. [49] found an association between the noise exposure and an increased heart rate, but the association became insignificant when PM_2.5_ was introduced to the model. At the population and city-level impact assessments, the benefits of cycling have clearly outweighed the negative effects of noise from cycling trips [3, see the reviews by 17].

### Greenery and cycling

Studies focusing on the linkages of greenery and cycling have mostly examined them from the perspective of cycling propensity and behaviour. The majority of studies find greenery to increase odds of cycling. In Barcelona, the amount of greenery near the study participants’ home or work location was positively associated with the commute by cycling [65]. Cycling propensity was also positively correlated with eye-level greenery in Hong Kong, but not with the bird-eye view greenery [66] highlighting the impact of the greenery measurement techniques. A study from Milwaukee found a consistent positive association between the street tree cover and the levels of active travel with walking and cycling combined [67]. A comparison study from multiple European cities found that the residents of neighbourhoods with more trees were more likely to cycle for transport [21]. In Beijing, travel satisfaction of cyclists was positively correlated with the level of travel environment greenery [32]. However, the number of urban parks in a city was not linked with the cycling levels in an international study that included fourteen cities from ten countries [68]. In route choice, greenery seems to play a role for cyclists. Some cyclists from the study population in Seattle favoured routes with street trees in a GPS-based study [69]. Similarly in Graz, cyclists were found to prefer routes with green and aquatic areas over the shortest possible routes [70] while in Berlin, respondents consistently preferred routes with higher levels of greenery [71]. The presence of greenery has also been found to connect with longer travel time [72]. Compared to studies focusing on travel behavioural connections of greenery and cycling, the presence of travel-related greenery during cycling and its physiological and mental impacts have received minimal attention. Focusing on the impacts of travel-related greenery, Cherrie et al. [73] and Zhang et al. [74], found a positive impact between the activity space exposure to green space and mental health in Edinburgh and Guangzhou respectively.

### Multiple exposures and cycling

Only a handful of studies have investigated simultaneous exposures in the cycling context, mostly from the perspective of air pollution and noise. While most studies have suggested moderate to weak positive correlation between air pollution and noise exposure on bicycle trips [59–61, 75], some studies have found stronger positive connections [76]. The variety of evidence likely stems from contextual, behavioural and meteorological conditions as well as a multitude of exposure measurement techniques [77]. To add complexity, cyclists’ perception may vary depending on the exposure. Ueberham et al. [56] studied cyclists’ perception to PNC,^2^ noise, and heat exposure in Leipzig and found that cyclists underestimated their PNC and noise exposure while heat exposure was estimated realistically. Marquart et al. [78] found in Berlin that green, blue and aesthetical landscapes contributed positively to perceptions of air pollution and noise exposure. This multifaceted complexity in studying multiple exposures and the difficulty of establishing causal relations has likely contributed to the lack of studies focusing on the health and well-being outcomes. However, an interesting non-cycling specific study was carried out by Roberts and Helbich [79] who focused on the relationship between multiple environmental exposures and depression symptoms along the participants’ residential location and daily mobility path. They found that exposure to green space near the residential address and along the mobility path was associated with a reduction in depressive symptoms, while blue space, noise, and air pollution had no association.

## Data and methods

### Data

#### Mobility

**Fig. 1:**
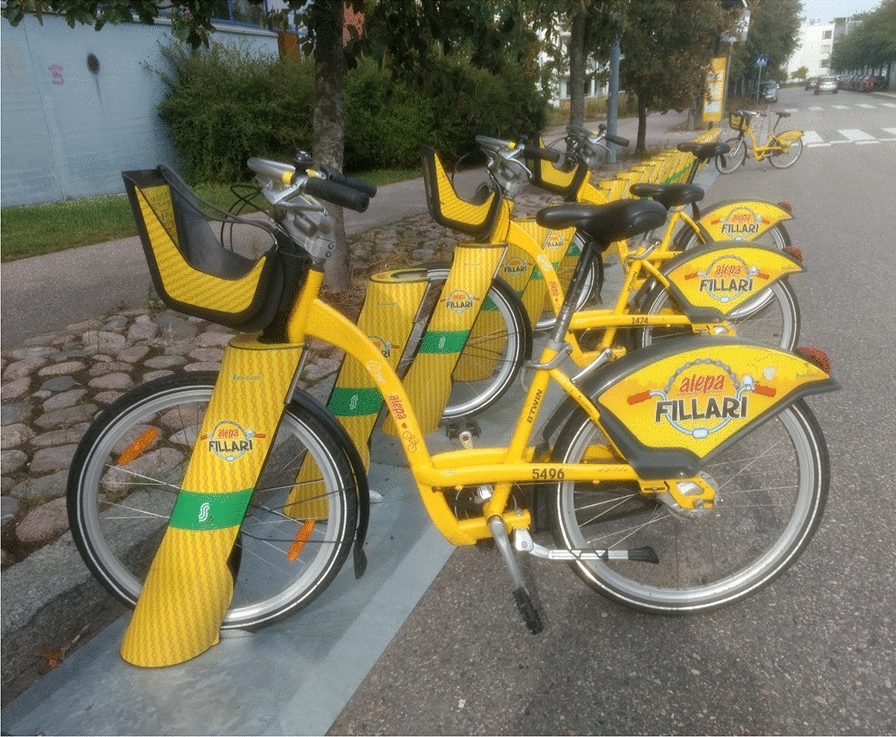
Example bikes of the Helsinki bike-sharing system. Photo by the authors

We used bike-sharing trip data from the local system to understand cyclists’ mobility in Helsinki, the capital of Finland. In a city with the population around of 630,000 inhabitants, the bike-sharing system has been actively used [34]. The system had 242 docking stations in 2019 and the area covered two-thirds of the city (see the example bikes and a station in Fig. 1). The data provided by the local system operator (Helsinki Region Transport—HRT) included all 3.1 million cycle trips by 61,300 distinct users between April and October 2019. Considering the number of trips and the wide spatial coverage of docking stations, we considered the bike-sharing system to be a spatially representative proxy for all cycling activity in the system area. The spatial distribution of bike-sharing trips is shown in Appendix A. The data variables included the origin and the return station and the start and the end time of each trip. To add the spatial context to the trips, we used another dataset provided by Helsinki Region Transport, which included the coordinate information of the bike-sharing system docking stations.

#### Exposure

For the air quality data, we used an annual average Air Quality Index (AQI) raster layer (spatial resolution 13 × 13 m) from Helsinki [80]. AQI is a composite index reaching from 1 to 5 (1 = good, 5 = very poor) and covering the concentrations of SO2, NO2, PM10, PM2.5, O3, CO and TRS,^3^ and which provides an overall characterisation of the actual air quality in the Helsinki Metropolitan Area. The Finnish Meteorological Institute (FMI) collects the raw data for the index at an hourly level through its monitoring network. The raw data is then coupled with the FMI-ENFUSER air quality model, which fuses historical measurement data, meteorological data, and GIS data on the environment to accurately represent air quality variation over the urban space and at fine scale [81].

The AQI index is health oriented. The values of each pollutant are compared to their class threshold values and the index at each hour is determined by the highest class, i.e., the worst performing concentrate [80]. With this, the AQI overcomes the challenge of using many individual pollutant variables while keeping health impacts relevant. In the annual data layer, the hourly AQI index values had been aggregated to represent the yearly average values. The Helsinki Region Environmental Services, who is the developer and maintainer of the data together with the Finnish National Institute of Health and Welfare, provided the annual data.

The noise data consisted of a vector layer of averaged day, evening, and nighttime A-weighted equivalent continuous sound pressure level (Lden) decibel values (dB) from road and rail traffic collected at 10 × 10 m spatial resolution. The data was provided by the Finnish Environment Institute [82] from 2017, and it had been collected in accordance with the EU Environmental Noise Directive 2002/49/EC.

The greenery data consisted of a Green View Index (GVI) layer for Helsinki developed by Toikka et al. [83]. The GVI layer modelled for the street network indicates the proportion of green vegetation visible at each street segment (from 0 to 1). The greenery values are based on Google Street View panoramas collected between 2009 and 2017 and sampled from every 20 m maximum distance, from which the street level greenery had been obtained with the MIT Treepedia deep learning algorithm [84]. The approach on obtaining GVI values from street view imagery was first proposed by Li et al. [85]. In areas where the street view images had not been available, the index had been supplemented with the regional land cover data from 2018 (HSY 2021) having the information of over two meters tall trees in the area. Example images of various GVI index classes from Helsinki are provided in Toikka [86]. The spatial distribution of AQI, average noise, and GVI in the study area are shown in Appendix B.

### Methodology

**Fig. 2:**
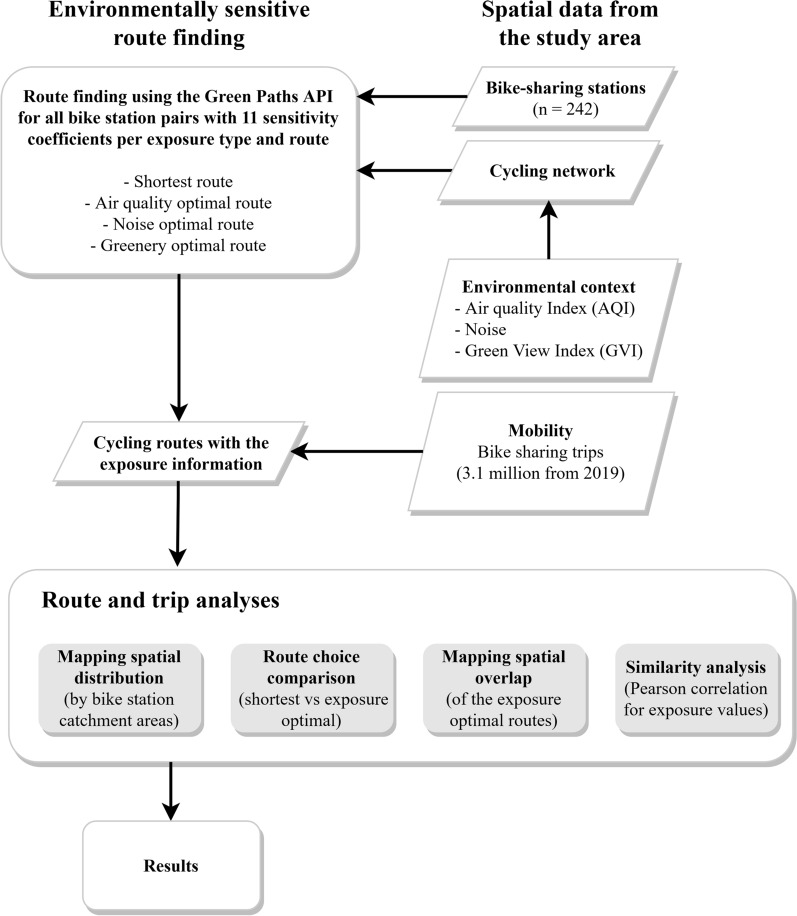
Flow chart of the analytical process

To demonstrate the feasibility of our approach, we carried out an exposure assessment in Helsinki, Finland (see the flowchart in Fig. 2). We used an in-house developed Green Paths Software, which finds exposure-optimal routes and provides information on exposure levels in Helsinki for walking and cycling based on air quality, noise, and greenery data [42]. To carry out the analysis at the population-level, we integrated spatially representative data on cycling to the software by using 3.1 million trips from the local bike-sharing system from 2019 and analysed the variation of three types of exposure both separately and together.

#### Exposure routing

Green Paths software [42] is a routing and exposure assessment tool. It is an open-source tool that has been built as a prototype for demonstrating path finding equipped with rich exposure metrics between selected origin–destination pairs. The software uses external exposure data on air quality, noise, and greenery (as described in 3.1) and applies least-cost routing with Dijkstra’s algorithm for route search. It uses OpenStreetMap walking and cycling street network data from the Helsinki region with the pre-calculated exposure cost attributes that are assigned to the network edges. To support environmentally sensitive routing, the software applies an environmental impedance function (EIF) to the routing algorithm, which by default is based on travel distance. The EIF enables the calculation of exposure-adjusted cost values [Eq. (1)]

1 $$Ce=Ct+Ct*ce*s$$

where $Ce$ is the total (composite) cost of the edge, $Ct$ is the base cost of the edge that is proportional to travel distance, $ce$ is an environmental cost coefficient and $s$ is an arbitrary sensitivity coefficient for assigning applicable weight(s) for the environmental cost component. The additional environmental cost is thus proportional to the value of the environmental variable and travel distance. The dynamic sensitivity coefficient *s* determines how much weight is given to the environmental cost coefficient, and this way enables the software to find multiple alternative routes for a single origin–destination pair. For route finding, the software provides an application-programming interface (API) where a user can input origin–destination pairs for routing requests and receive the shortest route and multiple alternative routes as the response that improve the trip exposure. Prior to finding routes, the application finds the nearest edges and nodes to the user-defined origin and destination locations. The complete technical documentation of the software can be found from Helle et al. [42].

Using the Green Paths API, we determined the shortest and three exposure-optimal routes for all the possible bike-sharing origin–destination pairs in Helsinki (n = 58,322). The shortest path routing is a standard approach in transport studies, and the shortest routes provided a standardised basis against which to compare the exposure-optimal routes. The exposure-optimal routing, on the other hand, provided a way to integrate environmental variables into route optimization. The exposure-optimal routes were identified for the origin–destination pairs by optimising air quality, noise, and greenery separately. The exposure-optimal route was defined as the route with the highest exposure reduction (air quality and noise) or increase (greenery) compared to the shortest route. For each exposure type and origin–destination pair, we carried out the routings with eleven sensitivity coefficients [0.1, 0.5, 1, 2, 5, 10, 100, 1000, 10,000, 100,000, and 1,000,000] since the varying spatial variation of the air quality, noise, and greenery required both smaller and higher sensitivity values. Because the different sensitivity coefficients resulted in multiple alternative routes for each exposure type, we compared the alternative routes against each other using their composite route cost, and with each exposure type selected the route with the lowest cumulative cost as the exposure-optimal route [see Eq. (1)]. The examination of the routing results confirmed that none of the exposure-optimal routes for any origin–destination pair had used the highest sensitivity coefficient, implying that we had found the optimal route for every pair. In the route selection, we applied a condition for the maximum detour distance for exposure-optimal routes, which was set to 15% based on Pritchard et al. [87]. These authors concluded that most studies on the route choice of cyclists have found that cyclists are on average willing to make 10–20% detours compared to the shortest route. Therefore, the exposure-optimal routes for all the comparative analyses between the shortest and the exposure-optimal routes were selected among the routes with less than a 15% detour.

#### Route and trip analyses

After identifying the shortest and the exposure-optimal routes for every origin–destination pair, we weighted the pairs with the bike-sharing system trip data to integrate information on realised cycling flows to the analyses. To map the spatial variation of exposures measured with AQI, noise, and GVI values, we created Voronoi polygons of the bike-sharing docking stations to identify the probable catchment area of each station and aggregated the exposure values to the polygons. Each Voronoi polygon represents the area from which the given station in the middle of the polygon is closer than any other station measured by Euclidean distance. We aggregated the average AQI, noise, and GVI exposure of all the trips based on the departure station to the polygons. Next, we compared the average exposure of the shortest and each exposure-optimal route and analysed the differences. We mapped the distribution of exposure values of all shortest and exposure-optimal routes and divided the exposure values into classes to measure the potential to improve exposure with a route choice in bike-sharing trips. We also analysed the distribution of the detour distances of the exposure-optimal routes to understand the distance trade-off in exposure improvement.

To understand the linkages of the AQI, noise, and GVI values in the bike-sharing routes, we further compared the spatial overlap of the exposure-optimal routes. With each origin–destination pair, we created a small buffer (5 m) for the shortest and the exposure-optimal route. Then, by overlaying the route buffer polygons, we calculated the proportion of the common route area between each exposure pair (AQI-noise, AQI-GVI, noise-GVI). Finally, we statistically analysed the linear correlation (Pearson) of the average AQI, noise, and GVI values along the bike-sharing routes to understand the relation of the exposure values at the route level. The data processing and the analytical workflow was carried out in Python 3.7 and the map visualisations in QGIS 3.14.

## Results

### Spatio-temporal distribution of exposures in bike-sharing trips

We found cycling trips in the study area to have good air quality (mean 1.91 AQI) (Table 1) with the mean value being below 2.0 AQI, which is the threshold for ‘good air quality’ [80]. However, the cyclists are exposed to high noise (mean 65.5 dB) and encounter low greenery (mean 0.17 GVI, i.e., 17% of visible greenery) during their cycling. On almost all the routes, the average noise exceeds the EEA 55 dB threshold for high daytime environmental noise [6]. Within the 15% detour distance threshold, an air-quality-optimal route is available for only 26.2% of the cycling trips, while a noise-optimal route is available for 78.3% and a greenery-optimal route for 47.4% of the trips. The results also demonstrate the value of including realised bike-sharing trip information in the analyses. For all the exposure types, the exposure improvement brought by the optimal route choice is lower when origin–destination pairs are trip-weighted instead of considering all the routes having equal importance. For greenery, the effect is the most significant, since the average trip-weighted exposure is 10 percentage points lower compared to the non-trip-weighted exposure.

**Table 1:** Descriptive exposure statistics (non-trip-weighted and trip-weighted) of bike-sharing trips in 2019 in Helsinki

Route	Bike-sharing routes (non-trip-weighted)				Mean exposure change of the optimal route (when available) compared to the shortest route	Mean distance (m)	Mean additional distance (m)
	Mean AQI^*^	Mean noise (dB)^**^	Mean GVI	Share of routes where exposure-optimal route is available within 15% detour distance			
Shortest	1.91	66.6	0.22	N/A	N/A	6211	N/A
AQI-optimal	1.89	64.1	0.22	54.3	− 0.02 (AQI)	6268	57
Noise-optimal	1.88	58.2	0.24	94.9	− 8.3 (dB)	6825	614
GVI-optimal	1.89	63.5	0.29	84.2	0.08 (GVI)	6547	336
	Bike-sharing routes (trip-weighted)						
Shortest	1.91	65.5	0.17	N/A	N/A	2067	N/A
AQI-optimal	1.90	64.0	0.18	26.2	− 0.01 (AQI)	2083	17
Noise-optimal	1.89	59.8	0.18	78.3	− 5.4 (dB)	2224	157
GVI-optimal	1.91	64.7	0.19	47.4	0.03 (GVI)	2116	50

*AQI* air quality index, *GVI* green view index

*Under 2.0 is the threshold for good air quality by national and EU standards [80]

**Over 55 dB 2.0 is the EEA threshold for high daytime noise [6]

**Fig. 3:**
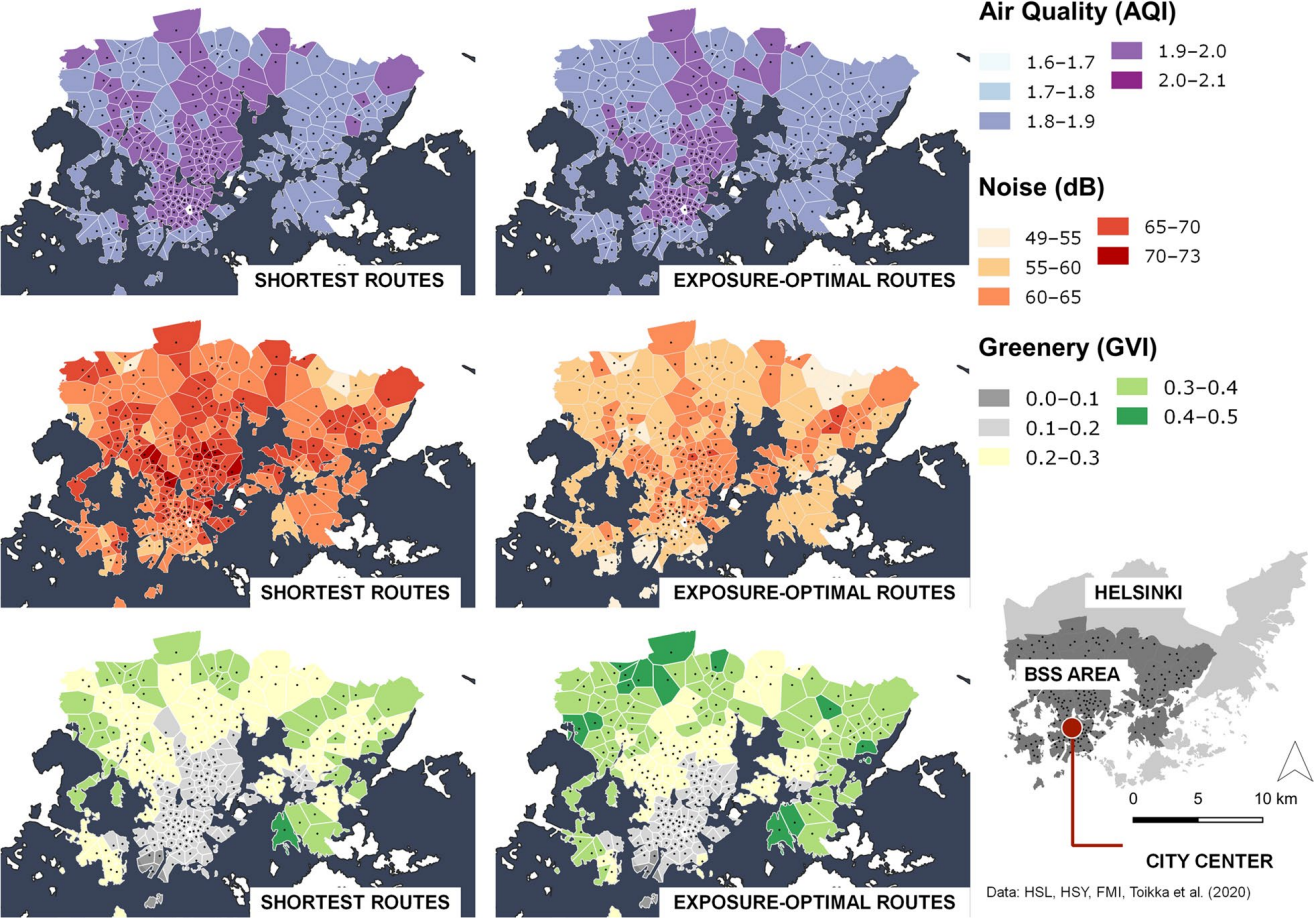
Spatial variation of average trip exposure to air pollution, noise, and greenery aggregated to the bike-sharing station catchment areas. Catchment area represents the area, in which the given station is closer than any other station measured by Euclidean distance. The maps on the left side show the variation for the shortest routes, the maps on the right side show the spatial variation for the exposure-optimal routes over the study area. The black dots on the map show the locations of bike-sharing docking stations

Figure 3 and the related difference maps (Appendix 3) show the spatial variation of air pollution, noise, and greenery exposure of the departing cycling trips over the study area. The values are aggregated to the catchment areas of the bike-sharing system docking stations. Throughout the area, air pollution exposure along the shortest routes and along the exposure-optimal routes has little variation (AQI varies on shortest routes from 1.83 to 1.97, on exposure-optimal routes from 1.83 to 1.94 AQI). Compared to air pollution, spatial variation of noise exposure is more evident (from 51.3 to 72.3 dB) with the highest noise exposure concentrations along the shortest routes located in central Helsinki. For the exposure optimal paths, the noise exposure is similarly distributed as it is along the shortest routes, but with consistently lower average noise values (from 49.9 to 67.6 dB). The greenery exposure, varying from 0.05 to 0.41 GVI along the shortest routes, is higher when starting the trip from the northern and eastern parts of the study area, while departing from the urban core results consistently in low green exposure (less than 0.2 GVI). The spatial variation of greenery exposure for the exposure-optimal routes is similar to the shortest routes (from 0.06 to 0.46 GVI) with an exception occurring in the northern and eastern parts of the study area. These routes not only have a higher greenery exposure on the shortest routes, but also possess higher potential for increasing greenery exposure on GVI-optimal route choice.

### Potential for exposure improvement with a route choice

**Fig. 4:**
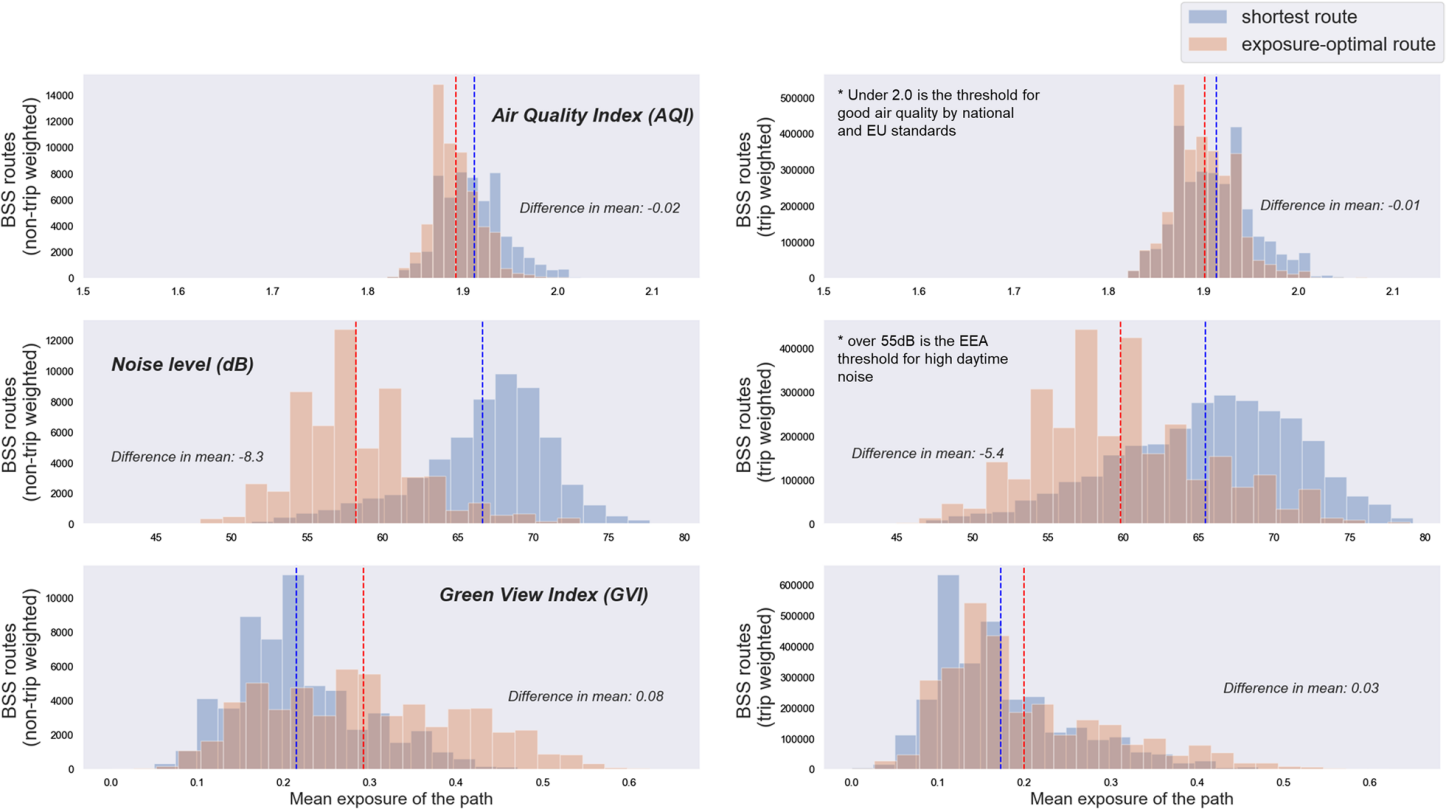
The distribution of air pollution, noise, and greenery exposure values for all bike-sharing system routes along the shortest and the exposure-optimal route. Non-trip-weighted distributions are shown on the left and trip-weighted on the right

The potential for improving environmental exposure whilst cycling in Helsinki is largest for noise reduction (Fig. 4). The impact of route choice for exposure improvement shows significant potential for noise reductions, with non-trip-weighted exposure decreasing noise an average of − 8.3 dB. The average non-trip-weighted potential for exposure improvement is also relatively large for greenery, being eight percentage points (0.08 GVI). However, when the routes are weighted by the number of cycling trips, the potential for both noise reduction and greenery increase is smaller (− 5.4 dB and 0.03 GVI). For noise, the potential is still substantial. Route choice does little to improve either the trip or non-trip-weighted air pollution exposure, with an average decrease of only − 0.01 and − 0.02 AQI respectively. The trip-level statistics on the potential of route choice to various exposure classes add to these findings (Appendix 4). Of the selected AQI classes, the change in time spent in the “AQI > 2” class appears largest, but is still small with an average decrease of only 0.6 min/trip, which is 4.3% of the average trip time. For noise exposure, the improvement potential is larger. The exposure optimal path reduces the noise exposure most to the “noise > 65 dB” class, which cyclists can decrease − 3.1 min/trip on average, which is 23.4% of the typical trip duration. For visible greenery, the average improvement potential is moderate with the largest increase of 1.1 min/trip to the “GVI > 0.2” class (i.e.,> 20% of visible greenery), which is 7.4% of the total trip time.

**Fig. 5:**
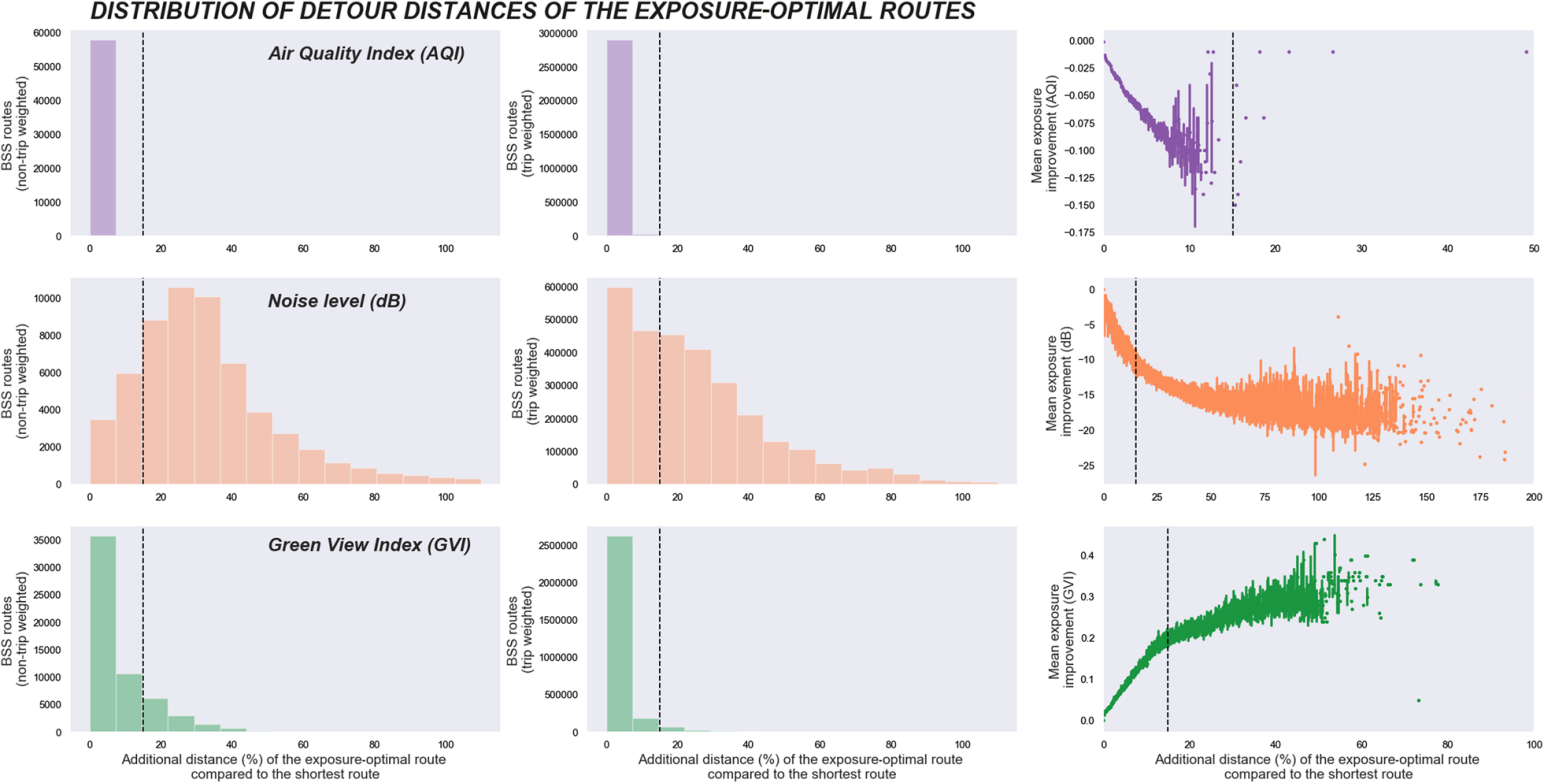
The distribution of detour distances (%) of the exposure-optimal routes compared to the shortest routes for all bike-sharing system origin–destination pairs. On the left, the charts show the distribution for non-trip-weighted routes, in the middle, the distribution for the trip-weighted routes is shown, and on the right, the improvements in average route exposures are plotted against the proportional detour distances. The bars display the detour distance distribution of all alternative exposure routes without any distance threshold, while the black dotted lines display the 15% detour distance threshold that we used in the comparative analyses in this study

To understand the potential of route choice in exposure improvement better, we analysed the detour distances between the shortest and the exposure-optimal routes and especially in relation to the 15% detour distance threshold that we used in the comparative analyses between the route options. Figure 5 shows that cyclists can reach the maximal air quality improvement in almost all origin–destination pairs (99.9%), both non-trip-weighted and trip-weighted, with no more than a 15% detour distance. However, as described earlier, the potential for AQI reductions is generally small in cycling trips in Helsinki and the proportion also includes those routes for which there is no alternative exposure-optimised route. The distribution of the detour distance for visible greenery is similar to air quality. In most origin–destination pairs (80.5%), cyclists can reach the maximal greenery increase within the 15% distance threshold limit and the proportion is even higher when the routes are trip-weighted by the cycling trips realised (96.0%). For noise exposure, the distribution is different. The route with the maximum noise reduction is available within the 15% detour distance threshold only in 16.9% of the non-trip-weighted, and in 37.0% of the trip-weighted origin–destination pairs. In longer detour distances, the curve begins to bend, implying that additional exposure gains of longer routes become smaller.

### Similarity of the exposure-optimal routes

**Fig. 6:**
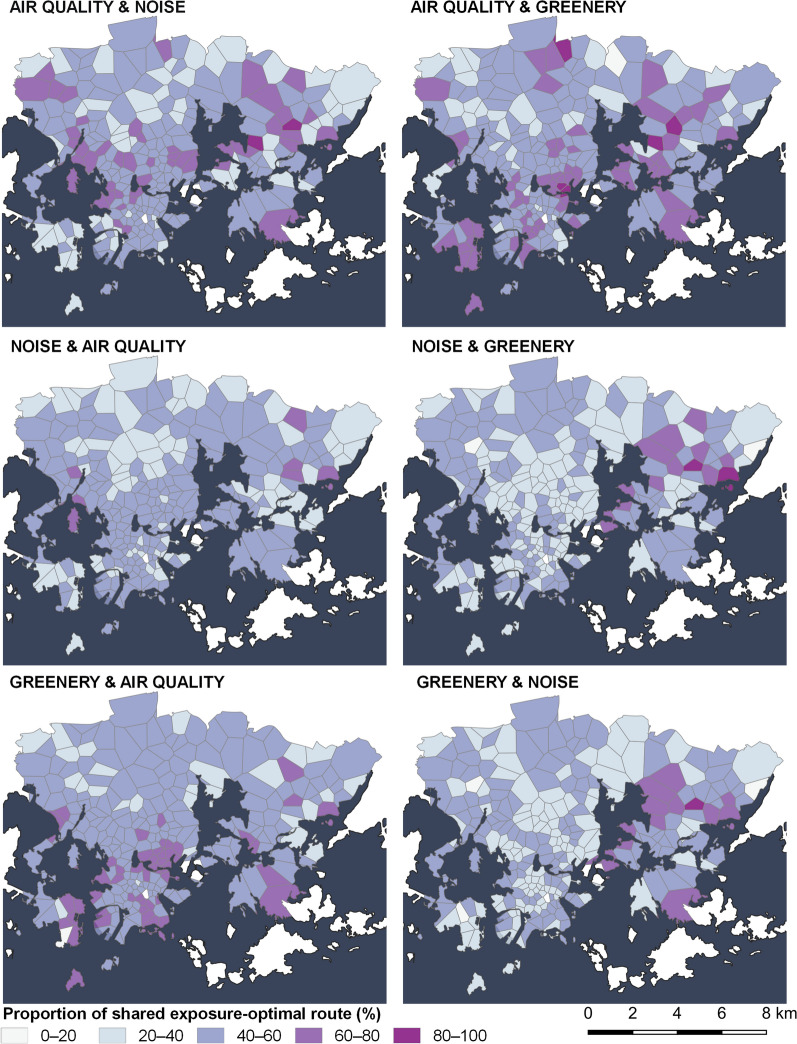
Average proportion of shared route between air quality, noise, and greenery-optimal routes (trip-weighted) over the study area. The common shares by route are aggregated to the bike-sharing station catchment areas

We further analysed the similarity of the exposure optimal cycling routes, both in terms of the shared route proportion and the correlation of exposure values. A few general trends can be observed in relation to the spatial similarity of the routes (Fig. 6). First, few areas in the study area, located in the Eastern Helsinki, consistently have a high proportion of the shared route between the air quality, noise, and greenery-optimal routes. This means that mostly in these areas it is possible to optimise all three exposures at once during cycling trips. In other words, in most parts of the study area, cyclists who want to improve their exposure must choose whether to emphasise air quality, noise, or greenery exposure. As the air quality exposure is more or less equal across the study area, the emphasis is more relevant in choosing routes that optimise noise or greenery. Secondly, air quality and greenery-optimal routes are more like each other compared to noise-optimal routes. On average, air-quality-optimal routes have 55.8% of the route distance in common with the greenery-optimal routes and 40.7% for the noise-optimal routes. When the origin–destination pairs are trip-weighted, the common share increases to 72.8% and 56.4% with greenery and noise, respectively. However, a significant reason for the higher commonality of air quality and greenery-optimal routes is the lack of alternative routes for these exposures (Appendix 5). This means that in many OD-pairs, there are few alternatives to the shortest route within the 15% detour distance threshold to improve the air quality and greenery exposure, while more alternatives exist for noise.

**Fig. 7:**
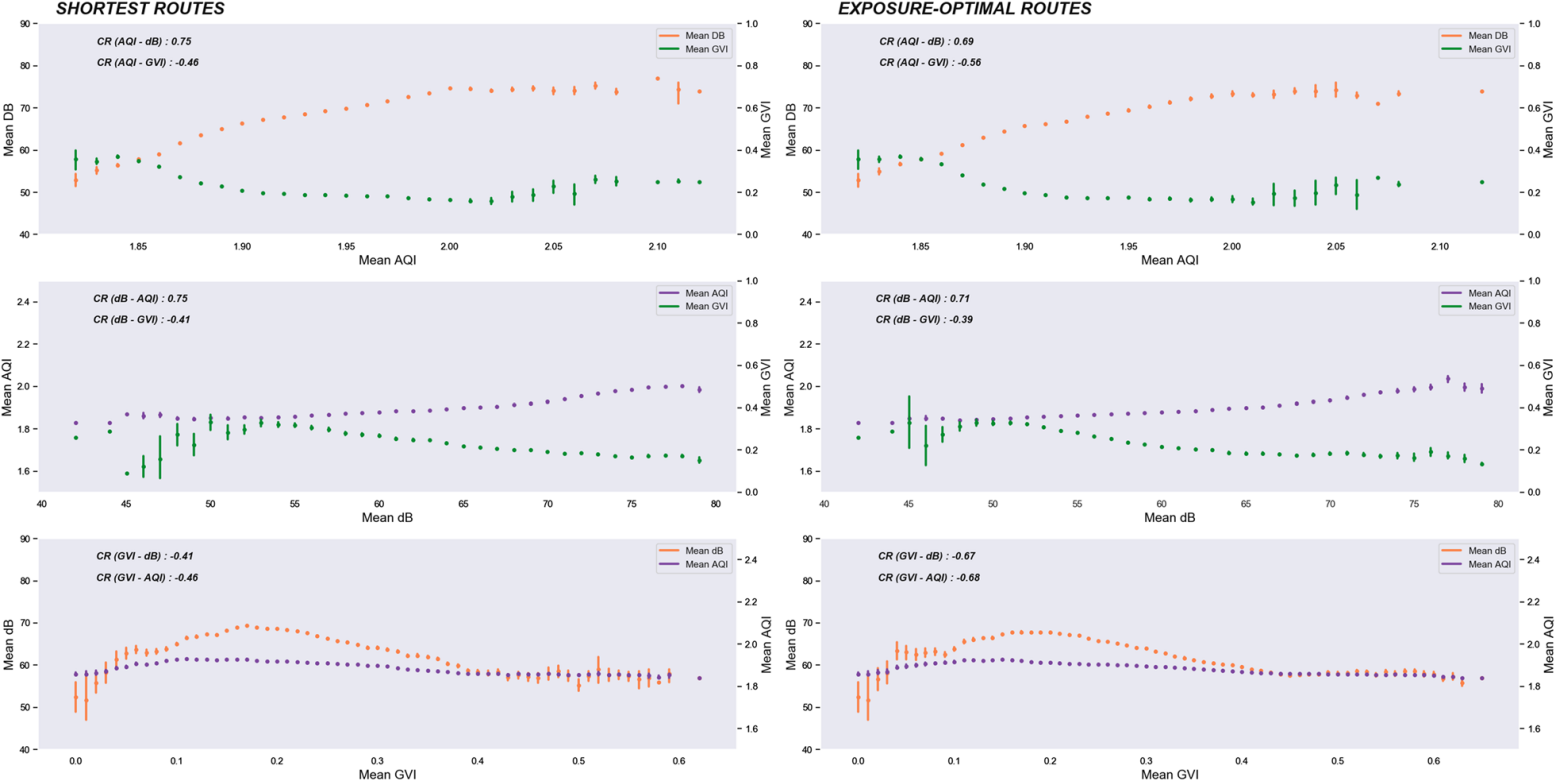
The relationship between air pollution, noise, and greenery values for bike-sharing system routes. On the left, the relationship along the shortest routes is displayed and on the right the relationship along the exposure-optimal routes. The exposure values are aggregated to the mean over the x-axis, and the 95% confidence interval is displayed as a line. In addition, the Pearson correlation coefficient is shown

The linear correlations between the route exposure values indicate logical connections between the selected exposures. Along the shortest routes the correlation between the AQI and noise values is positive and strong (R = 0.75) whereas between the AQI and GVI values, and the noise and GVI values the correlation is negative and less strong (R = − 0.46 and − 0.41, respectively) (Fig. 7). This means that when the air pollution increases, noise also increases, while the amount of visible greenery decreases. For the air-quality-optimal routes, the connection stays similar, as the correlation with the noise values is positive and strong (R = 0.69) and negative and less strong with the greenery values (R = − 0.56). For the noise-optimal routes, the correlation between the noise and AQI values is strong and positive (R = 0.71) and between the noise and GVI values moderate and negative (R = − 0.39). For the greenery-optimal routes, the correlation of GVI with the AQI and noise values is notably stronger compared to the shortest routes (R = − 0.68 and R = − 0.67 respectively), which further points to the direction that greenery increases connect with lower noise and air pollution in Helsinki. The shapes of the regression plots indicate that while noise and air pollution increase together linearly, average greenery plateaus at levels around 0.15 GVI (i.e., 15% of visible greenery) even if noise pollution or air pollution increase. At the other end of the greenery distribution, the decrease of the air pollution and noise levels seems to plateau when GVI reaches 0.4.

## Discussion

The increased availability of spatial data and the emergence of tools for environmentally-sensitive route planning have provided new opportunities for exposure research and urban studies. As demonstrated by this study, the integration of these data and tools enables researchers and planners to carry out population-level analyses on the spatio-temporal distribution of various types of environmental exposure and to identify areas where travellers’ exposure exceeds safe levels. This has a significant value for the efforts to mitigate large negative health impacts of daily travel, promote active travel, and provide equal access to healthy travel infrastructure in cities. The value of our approach is also underlined by the increased understanding how travel satisfaction and attractiveness of travel environments play a key role in travel decisions [23, 88]. Conceptually, approaches like ours are a step towards giving a greater emphasis to the positive utility of travel as well as to the restorative qualities of the travel environment. Only more recently, has there been a growing interest toward these factors in transportation research and planning, in which the endeavour for travel time minimisation has typically prevailed [89–92]. Our approach also directs more attention to natural environment qualities as contributors of good travel environments as most walkability and bikeability studies have been more focused on the impact of built environment [e.g. 21, 93, 94].

The findings of our Helsinki case study show that the noise exposure of cyclists in Helsinki significantly exceeds the official guidelines throughout the study area. This is in line with the previous literature, which found cyclists to be exposed to excessive noise levels in large cities [59, 60]. For the air pollution exposure, the average levels in almost all our routes were within the good air quality category. The air quality levels likely illustrate the characteristics of Helsinki, which is among the least polluted cities in Europe [95]. While similar guidelines for the urban greenery are currently lacking, MIT Treepedia [96] provides a greenery comparison of 31 selected global cities contextualising our values. In the comparison, the greenery levels vary from 0.09 to 0.36 GVI, with the average being 0.20 GVI, which places the average greenery of cycling trips in our study (0.17 GVI) at the lower end of the spectrum.

These results also demonstrate the need to include data on realised travel behaviour to exposure measures. With each of the three exposure types, the potential to improve exposure load with alternative route choice appears smaller in Helsinki when the routes are weighted by realised mobility data. In our study area, especially greenery exposure is a good example of this. While Helsinki as a whole is situated at the upper end in the international greenery comparison (median 0.32 GVI, Toikka [86]), the greenery is mostly located outside the urban core. Since the majority of bike sharing trips that we used as a proxy for cycling occur in the urban core areas, these cyclists encounter low greenery despite the higher total greenery in the city. While there is some uncertainty in how well the bike-sharing trips represent overall cycling patterns, these systems provide useful data on realised travel behaviour for active travel-oriented exposure assessments. This is because of their ubiquity in cities, typically large coverage areas, good temporal coverage, and popularity among citizens [34].

In respect of the impacts of alternative route choice, we show that it can be an effective way for cyclists to improve their travel-related exposure with a modest increase in travel distance. However, the importance of route choice significantly varies both spatially and by exposure type. In our study area, the exposure-optimal routes provided considerable potential for noise reduction for cyclists, but only a little for improved air pollution or greenery exposure. While our result might be sensitive to the local context, they follow previous studies that have found route choice to be a functional way to mitigate air pollution and noise exposure while walking or cycling [97, 98]. Obviously, environmental exposure is only one of the many factors that cyclists’ may want to optimise in their route-choices. Previous literature has extensively studied important built environment factors behind route choices [e.g. 21]. However, the impact of natural environment factors is less studied. Integrating such variables into walkability and bikeability indices holds potential for further understanding, which kind of urban travel environments attract walking and cycling.

Our results on the interlinkages between the three exposure types revealed that trip-level air pollution and noise exposure seem to follow each other in Helsinki while greenery exposure is negatively, but less strongly, correlated with the other two exposure types on cycling trips. Some previous studies have found similar evidence on the relation of air pollution and noise [60, 76], but also that even high noise or air pollution exposure can be perceived positively near green or aesthetic landscapes [78]. Capturing these types of spatial linkages between multiple exposures can help in understanding the co-existence and mutual dependence of various environmental qualities, being a step toward assessing their cumulative health impacts, which are currently little known [11].

There are some limitations in the proposed analytical approach. Firstly, in the absence of real route information, we modelled the use of the shortest routes by cyclists. We acknowledge that individual differences in route preferences exist, which adds spatial uncertainty to the results. However, the shortest path routing is a standard approach in transport modelling. The modelled shortest routes provided a standardised basis for the comparative route analysis, showing the true effect of informed route choice in exposure optimisation. Secondly, common to the environmental exposure studies, a lack of temporal data is another source of uncertainty. We used the best available annual average layers on each exposure, but the annual layers might flatten daily, monthly, and seasonal variation, thus affecting our results. For example, the air quality levels may be higher in the springtime due to higher levels of road dust, or the noise levels might have significant hourly variations. Furthermore, the greenery levels only describe the situation during the time of the year when the trees have leaves and weather at the time of street-view image collection might influence the greenery values. However, as our approach is intended to model aggregate patterns and spatial variation in travel-related exposure over a long period of time and at population level, the impact of these temporal fluctuations is likely weaker. Thirdly, we created the catchment areas for the bike-sharing stations using Euclidean distance, not network distance. While this adds some uncertainty, the benefit of this approach was its lightness, which supports the applicability to other contexts. Euclidean and network catchments also have typically high mutual correlation [99]. Finally, a robust understanding of the health and wellbeing impacts of various greenery levels encountered during travel and measured with GVI is still mostly lacking from existing literature, which limits our conclusions on the importance of cyclists’ greenery exposure.

To advance the research field further, there is a need for more detailed exposure measures to improve the exposure estimates and the sensitivity of route planning tools. Low-cost portable exposure sensors could provide spatially and temporally more precise information on the variation of air quality and noise levels, for example inside the street canyons and over the course of the day [100]. In addition, there is a need to advance open data practises for better availability of comparable mobility and environmental data. This would also facilitate the inclusion of less studied exposure types such as greenery or heat to exposure assessments, as well as comparative analyses between cities. For example, in our study area, more health-relevant pollutants could be added to the AQI [101]. Better exposure measures for their part would facilitate research on the health and wellbeing outcomes related to travel-related exposures. Currently, few studies have linked the exposure estimates to measured health outcomes [11]. Further research on the importance of route choice is also needed. It could help to understand short-term and long-term health impacts of environmentally favourable routes in various contexts, but also to gain information on which exposures different travellers prefer to avoid or to increase if given the choice and the necessary exposure information. Finally, it is crucial to advance understanding on the equity of travel-related exposure between areas and socio-demographic groups. Our study demonstrates how the travel-related exposure of cycling trips may significantly vary over the urban area. Linking this information to socio-economic information is needed to support planning for the provision of equitable access to healthy travel environments for everyone.

## Conclusions

This study provides an analysis framework to measure multiple forms of environmental exposure during cycling at population-level. The proposed approach provides a novel contribution to the environmental exposure literature. Specifically, it contributes to understanding of urban scale variation and linkages of multiple exposures, as well as the potential of route choice in improving travel-related exposure of cycling. Since cycling and walking often use the same networks, the approach may be applicable to active travel more broadly, which highlights its value. Reliance on open sources tools and data also increases transferability to other cities. In all, the analysis framework supports cities in their efforts to mitigate the negative health effects of travel while increasing the share of active travel.

## Figures and Tables

**Fig. 8:**
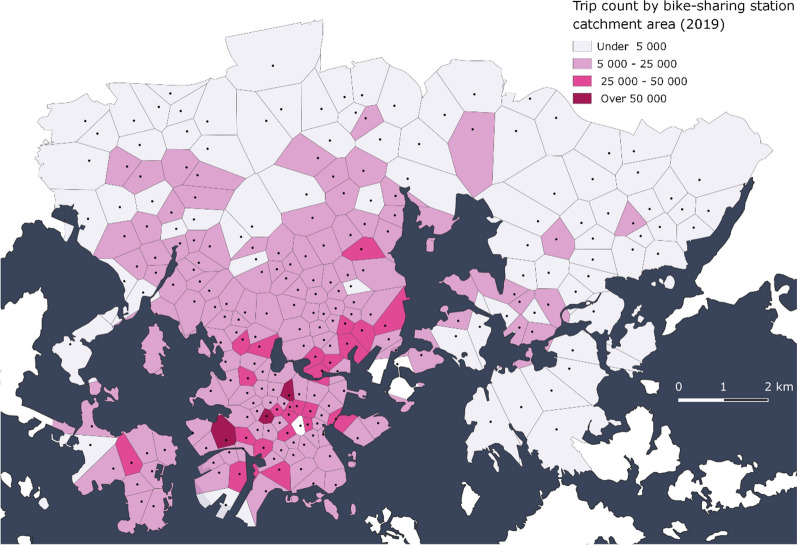
The count of departing bike-sharing trips by the bike-sharing docking station catchment area in 2019 in Helsinki. The catchment areas are delineated with Voronoi polygons

**Fig. 9:**
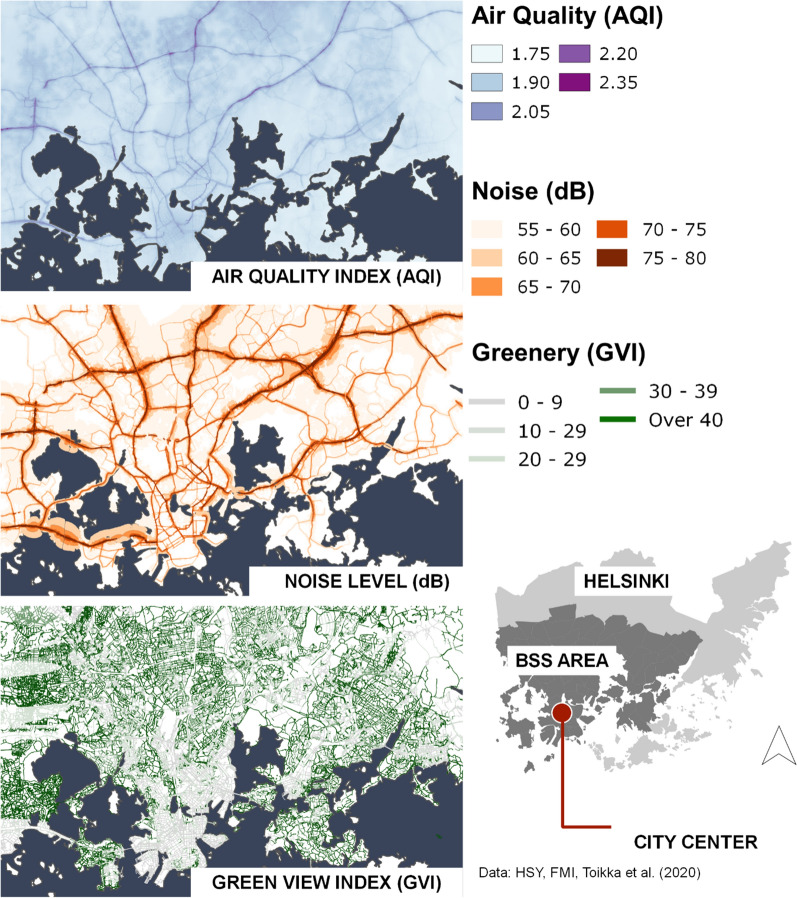
The distribution of Air Quality Index (AQI), noise and Green View Index (GVI) values over the study area

**Fig. 10:**
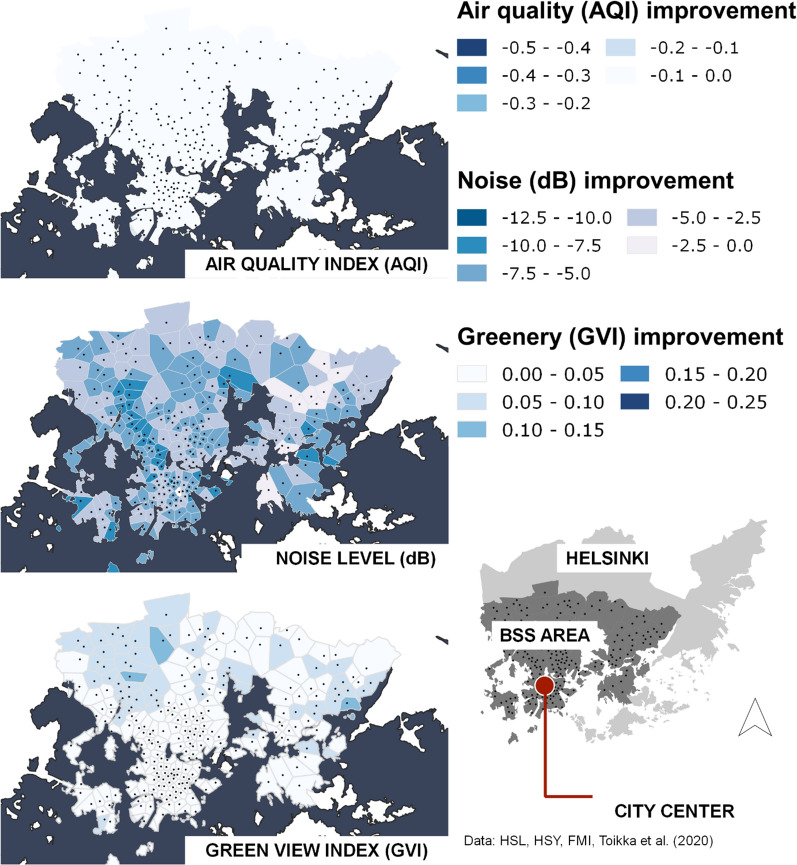
The spatial variation in opportunities to improve air quality, noise, and greenery exposure with a route choice in cycling trips. The values, which are aggregated to the bike-sharing station catchment areas, represent the average difference (trip-weighted) in air quality, noise, and greenery exposure between the shortest route and the exposure-optimal route. The black dots on the map show the locations of bike-sharing stations

**Fig. 11:**
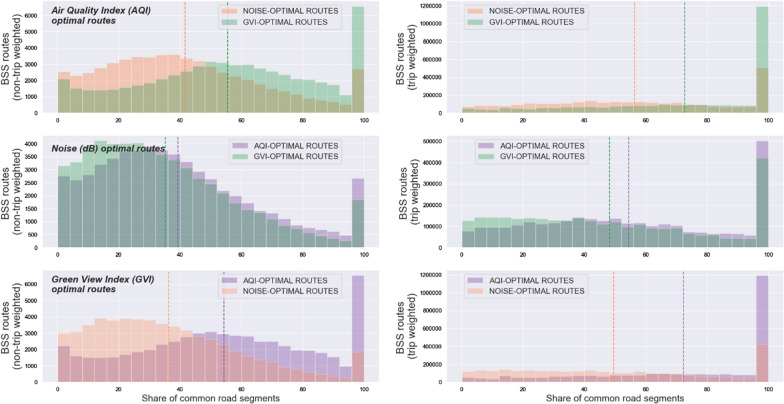
The distribution of shared route percentage between the air quality, noise, and greenery optimal routes for all origin–destination pairs. The charts on the left show the non-trip weighted distribution and the charts on the right show the trip-weighted distribution of shared route percentage. The highest bars at the ends of the x-axis correspond to the situation where exposure-optimal route is not available for the given origin–destination pair and therefore the shortest route is used

## Data Availability

The source code of the route planning and exposure assessment software used in this study is publicly accessible at https://github.com/DigitalGeographyLab/green-path-server. The bike-sharing dataset analysed during the current study is available in the Helsinki Region Transport repository at https://hri.fi/data/en_GB/dataset/helsingin-ja-espoon-kaupunkipyorilla-ajatut-matkat The air quality dataset analysed during the current study is available in the FMI repository at https://en.ilmatieteenlaitos.fi/open-science-research-data The noise dataset analysed during the current study is available in the Finnish Environment Institute repository at https://ckan.ymparisto.fi/dataset/ymparistomeludirektiivin-mukaiset-melualueet-2017 The greenery dataset analysed during the current study has been published by the authors and available at https://www.sciencedirect.com/science/article/pii/S2352340920304959

## See Table 2

**Table 2:** Descriptive statistics for the change in trip time to various exposure classes when selecting the exposure-optimal route over the shortest route in bike-sharing trips in Helsinki

Change in exposure to	AQI over 1.5	AQI over 2.0	AQI over 2.5	AQI over 3.0	Noise over 55 db	Noise over 60 db	Noise over 65 db	Noise over 70 db	GVI over 20	GVI over 30	GVI over 40	GVI over 50
Mean (min/trip)	− 0.9	− 0.6	0	0	− 1.9	− 2.7	− 3.1	− 2.5	1.1	0.9	0.7	0.4
Median (min/trip)	0	0	0	0	− 0.9	− 1.5	− 1.8	− 1.1	0.0	0.0	0.0	0
STD (min/trip)	0.23	1.56	0	0	2.6	3.4	3.9	3.6	2.1	2.3	2.0	1.5
Relative change (% of trip time)	− 0.6	− 4.5	0	0	− 14.0	− 20.2	− 23.4	− 18.1	7.4	6.8	4.7	2.6

## Abbreviations

API: Application programming interface
AQI: Air quality index
CO: Carbon monoxide
EEA: European Environmental Agency
EIF: Environmental impedance function
FMI: Finnish Meteorological Institute
GVI: Green view index
GPS: Global positioning system
Lden: The A-weighted, Leq (equivalent noise level) over a whole day
NO_2_: Nitrogen oxide
O_3_: Ozone
PM_2.5_: Fine particulate matter with a diameter of 2.5 µm (μm) or less
PM_10_: Coarse particulate matter with a diameter of 10 µm (μm) or less
PNC: Particle number concentration (PNC)
SO_2_: Sulphur dioxide
TRS: Total reduced sulphur compounds
UFP: Ultrafine particles (UFP)
UN: United Nations

## Footnotes

1 Particulate Matter (PM), Ultrafine Particles (UFP), Particle Number Concentration (PNC).

2 Particle Number Concentration (PNC).

3 Sulphur dioxide (SO2), Nitrogen oxide (NO2), Particulate matter (PM), Ozone (03), Carbon monoxide (CO), Total reduced sulphur compounds (TRS).
